# Loss of maternal *Trim28* causes male-predominant early embryonic lethality

**DOI:** 10.1101/gad.291195.116

**Published:** 2017-01-01

**Authors:** Abhishek Sampath Kumar, Michelle K.Y. Seah, Ka Yi Ling, Yaju Wang, Joel H.L. Tan, Sandra Nitsch, Shu Ly Lim, Chanchao Lorthongpanich, Heike Wollmann, Diana H.P. Low, Ernesto Guccione, Daniel M. Messerschmidt

**Affiliations:** 1 Developmental Epigenetics and Disease Group, Institute of Molecular and Cell Biology (IMCB), Agency for Science, Technology, and Research (A*STAR), Singapore 138673;; 2 Department of Biochemistry, Yong Loo Lin School of Medicine, National University of Singapore, Singapore 119074;; 3 Siriraj Center of Excellence for Stem Cell Research, Mahidol University, Bangkok 10700, Thailand;; 4 Next-Generation Sequencing Unit of DNA Sequencing Facility, IMCB, A*STAR, 138673, Singapore;; 5 Methyltransferases in Development and Disease Group, IMCB, A*STAR, Singapore 138673

**Keywords:** DNA methylation, epigenetics, Rbmy, reprogramming, splicing, Trim28

## Abstract

Global DNA demethylation is a hallmark of embryonic epigenetic reprogramming. However, embryos engage noncanonical DNA methylation maintenance mechanisms to ensure inheritance of exceptional epigenetic germline features to the soma. Besides the paradigmatic genomic imprints, these exceptions remain ill-defined, and the mechanisms ensuring demethylation resistance in the light of global reprogramming remain poorly understood. Here we show that the Y-linked gene *Rbmy1a1* is highly methylated in mature sperm and resists DNA demethylation post-fertilization. Aberrant hypomethylation of the *Rbmy1a1* promoter results in its ectopic activation, causing male-specific peri-implantation lethality. *Rbmy1a1* is a novel target of the TRIM28 complex, which is required to protect its repressive epigenetic state during embryonic epigenetic reprogramming.

## Abstract (precis)

Kumar et al. show that the Y-linked gene *Rbmy1a1* is highly methylated in mature sperm and resists DNA demethylation post-fertilization. Aberrant hypomethylation of the *Rbmy1a1* promoter results in its ectopic activation, causing male-specific peri-implantation lethality.

Germ cells are highly differentiated cells that give rise to the next generation's embryo upon fertilization. Their unique epigenome, which reflects their specialization, must be reprogrammed for proper development. A hallmark of this process is genome-wide DNA demethylation, culminating in the open chromatin state of the epiblast (Messerschmidt et al. 2014). However, specialized genomic regions must retain DNA methylation for inheritance of vital epigenetic germline features to the soma. Prominent among them are genomic imprints, which engage noncanonical DNA methylation maintenance mechanisms to become reprogramming-resistant. Despite the importance of this process, only a few reprogramming-resistant regions and their functions have been described beyond genomic imprints, and the mechanisms allowing methylation maintenance in light of epigenetic reprogramming are poorly understood (Branco et al. 2016).

In early embryos, maintenance DNA methyltransferase (DNMT1) levels are low to facilitate global demethylation, yet methylation is still maintained by DNMT1 at imprinted regions (Hirasawa et al. 2008). Our previous work revealed a fundamental role of maternal TRIM28 in this process, achieved through TRIM28's recruitment by the Krueppel-associated box domain zinc finger protein (KRAB-ZFP) ZFP57 and noncanonical targeting of DNMT1 (Messerschmidt et al. 2012; Lorthongpanich et al. 2013).

Paralleling the imprinting defects in maternal *Trim28* mutant embryos, we now expose a sex-specific early embryonic lethality phenotype. Our new findings show that, besides imprints, TRIM28 safeguards germline-to-soma inheritance of epigenetic features at other genomic regions in an exquisitely stage-dependent manner.

## Results and Discussion

TRIM28 is essential for development and maternal or zygotic deletion (Supplemental Fig. S1A) and is embryonic-lethal (Cammas et al. 2000; Messerschmidt et al. 2012). In zygotic mutants, maternally inherited *Trim28* gene products remain unperturbed, and embryos arrest at gastrulation. Removal of maternal *Trim28* also results in embryonic lethality; however, timing and causality are remarkably variable, presumably owing to the mosaic nature of DNA methylation defects leading to variable gene expression (Messerschmidt et al. 2012; Lorthongpanich et al. 2013).

**Figure 1.**
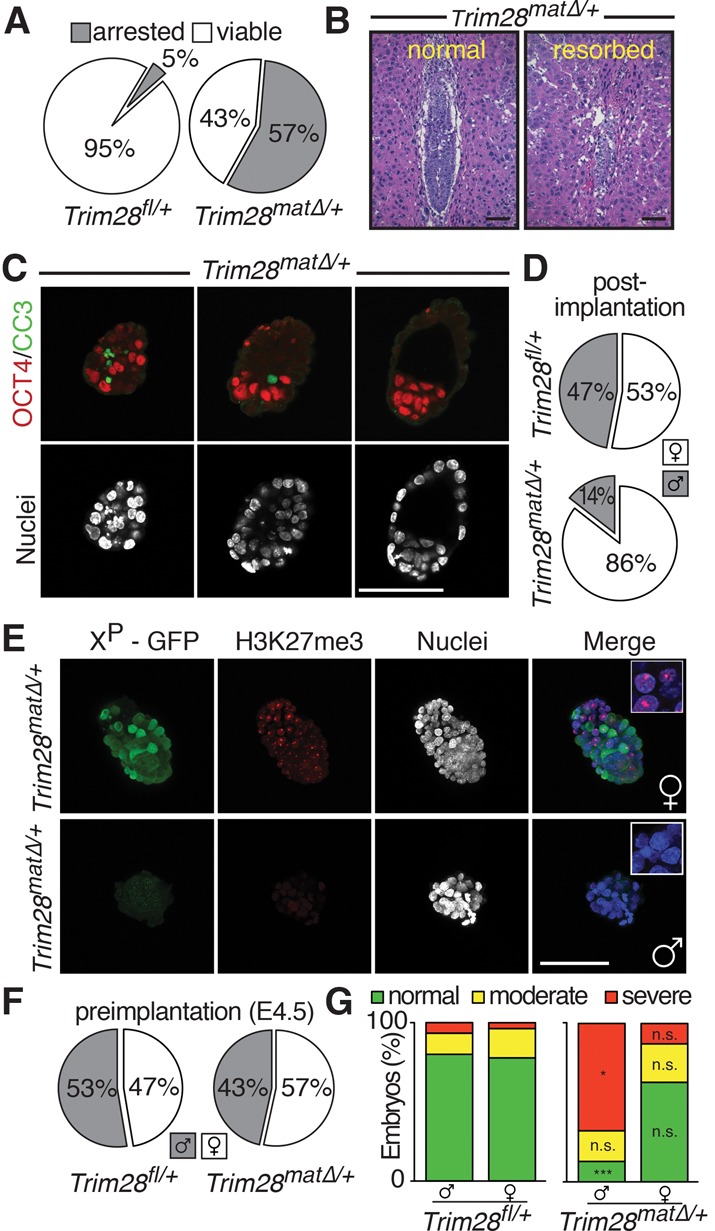
The absence of maternal *Trim28* causes male-predominant early embryonic lethality. (*A*) Percentage of post-implantation embryonic lethality in *Trim28*^f/+^ and *Trim28*^*mat*^^Δ/+^ embryos. (*B*) H&E-stained sections of deciduomas of normal and resorbed *Trim28*^*mat*Δ/+^ littermates at embryonic day 6.5 (E6.5). (*C*) OCT3/4 and cleaved Caspase 3 (CC3) immunofluorescence staining of E4.5 *Trim28*^*mat*^^Δ/+^ littermates. (*D*) Observed sex ratio in post-implantation mutant and control litters. (*E*) Female (*top*) and male (*bottom*) E4.5 *Trim28*^*mat*^^Δ/+^ embryos showing normal and abnormal morphology, respectively. (*F*) Observed sex ratio in preimplantation mutant and control litters. (*G*) Quantification of the morphological defects observed in mutant/control male/female embryos. Bars, 100 µm.

Despite the stochastic nature of the phenotype, we found 57% (*n* = 252 out of 444) of maternal-null *Trim28* (*Trim28*^*mat*Δ/+^) embryos to be resorbed immediately after implantation (Fig. 1A,B; Supplemental Fig. S1B; Messerschmidt et al. 2012). This means that *Trim28*^*mat*Δ/+^ blastocysts form functional trophectoderm (TE) and induce decidualization, yet merely half are capable of further development. In comparison, only 5% (*n* = 7 out of 142) of control (*Trim28*^*f*/+^) embryos were found resorbed (Fig. 1A).

Preimplantation *Trim28*^*mat*Δ/+^ embryos are unperturbed (Messerschmidt et al. 2012). We therefore examined implanting embryonic day 4.5 (E4.5) embryos, finding variable degrees of morphological abnormalities among littermates. Abnormal embryos displaying fragmented, pyknotic nuclei coinciding with active Caspase 3 staining were found next to normal, expanded blastocysts (Fig. 1C). However, despite being morphologically abnormal, these embryos still showed normal lineage segregation, displaying inner cell mass (ICM) and TE markers (Fig. 1C; Supplemental Fig. S1C).

The frequency of peri-implantation lethality suggests a close to 1:1 segregation ratio of the phenotype despite embryos being genetically identical (Supplemental Fig. S1A). Furthermore, the *Trim28*^*f/f*^ line was extensively backcrossed to the C57BL/6J genetic background, excluding a segregation of strain-specific determinants (Cammas et al. 2000; Messerschmidt et al. 2012). Instead, sex determination of embryos surviving beyond implantation revealed a remarkable sex ratio bias, with 86% (*n* = 65 out of 76) of the surviving *Trim28*^*mat*Δ/+^ embryos being female, suggesting a gonosome-linked phenotype. Control litters segregated as expected in a close to 1:1 ratio (53% [*n* = 27] females and 47% [*n* = 24] males) (Fig. 1D).

For faithful, noninvasive sexing of embryos, we used an X-linked GFP reporter (Supplemental Fig. S2; Hadjantonakis et al. 1998). We excluded *Trim28* mutant-related loss of GFP expression in females by examining embryos for the presence or absence of typical punctate H3K27me3 staining labeling the inactivated X chromosome and conducted *Sry* genotyping and/or *Xist* expression analysis (Fig. 1E; data not shown). While females always showed reliable GFP expression, male embryos remained GFP-negative, and X inactivation was not evident at E4.5. In contrast to post-implantation stages, the sex ratio remained balanced at E4.5, with 47%/53% (*n* = 57) females and males in control and 57%/43% (*n* = 156) females and males in mutant litters, respectively (Fig. 1F). However, when categorized morphologically (Supplemental Fig. S1D), a significant increase of severely defective mutant males was observed, while mutant females showed no significant changes in morphological categorization (Fig. 1G). Thus, the absence of maternal TRIM28 causes male-predominant peri-implantation embryonic lethality.

Sex-specific differences in mouse preimplantation embryos are limited to gonosomes, including X-chromosome dosage compensation in females. We found no indication of the characteristic H3K27me3 labeling of condensed X chromosomes in mutant males or a second condensed X chromosome in female cells, eliminating aberrant “imprinted” maternal X inactivation, possibly caused by exposure of the maternal X chromosome to the *Trim28*-null environment in the oocyte (Fig. 1E, insets; Supplemental Fig. S2B,C). Post-implantation *Trim28*^*mat*Δ/+^/XX^P^-GFP females displayed GFP-negative and GFP-positive cells at comparable ratios (Supplemental Fig. S2), excluding the specific loss of cells relying on the maternally inherited, potentially “defective” X chromosome (50% of cells in females and all cells in males).

**Figure 2.**
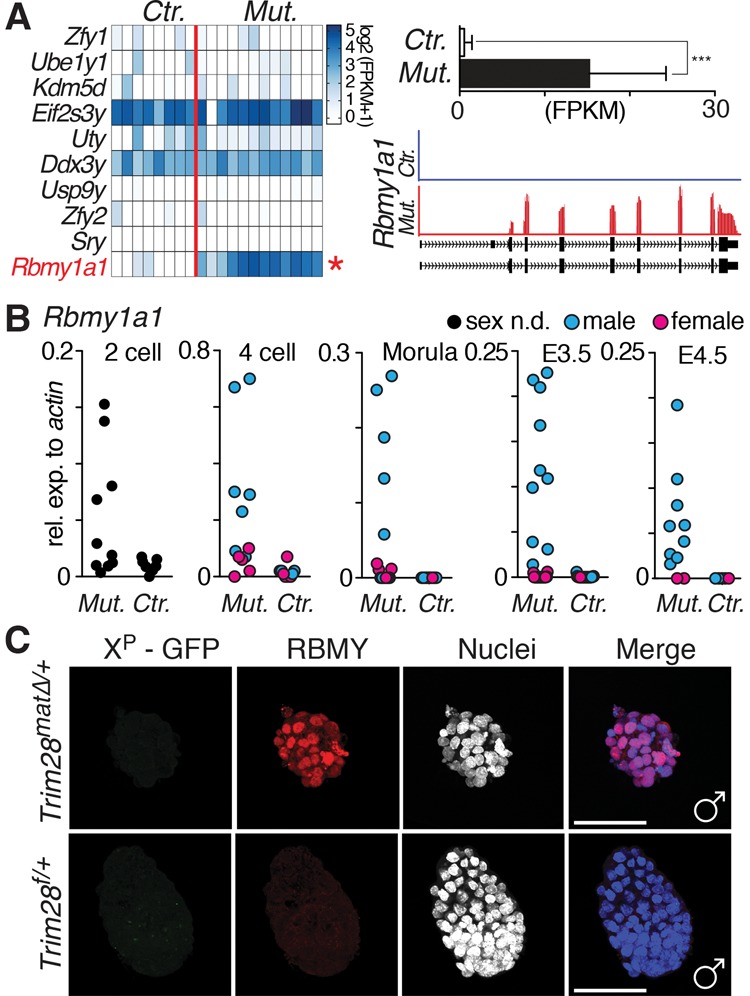
Male-specific transcriptional changes in *Trim28*^*mat*^^Δ/+^ embryos. (*A*) RNA sequencing (RNA-seq) of individual mutant and control E3.5 blastocysts. The heat map represents log_2_ (FPKM+1) values of Y-chromosome-encoded genes. (*B*) qRT–PCR analysis of *Rbmy1a1* expression in preimplantation embryos from the two-cell to the late blastocyst stage in mutants and controls. Sex was not determined by *Xist* or X-GFP expression (not possible at the two-cell stage). (*C*) Immunofluorescence staining for RBMY1A1 in mutant and control E4.5 male embryos. Bar, 100 µm.

Unable to find global X-linked defects, we analyzed gene expression in mutant (*n* = 12) and control (*n* = 7) blastocysts (Supplemental Table 1). Sixty-seven and 68 transcripts were up-regulated and down-regulated, respectively, clustering in 16 gene ontology categories (Supplemental Tables 2–3). X-linked genes were not enriched; five transcripts were moderately down-regulated, and one transcript was weakly induced. In contrast, one Y-linked transcript (*Gm10352* or *Rbmy1a1*) was highly induced in mutants and virtually absent in controls. Exposure of the paternal genome to the *Trim28*-null environment after fertilization causes DNA demethylation of paternal imprints (Messerschmidt et al. 2012; Lorthongpanich et al. 2013). The absence of maternal TRIM28 may also relieve gene repression through hypomethylation. Focusing on the Y chromosome, we found that, other than *Rbmy1a1*, which showed a highly significant activation in mutants, other Y-linked genes are not expressed at all or not differentially expressed in mutants and controls (Fig. 2A). *Rbmy1a1* is a multicopy gene (∼30 copies) (Soh et al. 2014) encoding a testis-specific RNA-binding protein involved in alternative mRNA splicing (Zeng et al. 2008). Nine copies are reliably annotated (GRCm38/mm10) and encode for the full *Rbmy1a1* ORF. For subsequent analyses, we therefore selected promoter and coding regions that are conserved among all annotated copies. Quantitative RT–PCR (qRT–PCR) analysis throughout preimplantation confirmed the dramatic *Rbmy1a1* activation in individual *Trim28*^*mat*^^Δ/+^ embryos (Fig. 2B), occurring as early as the two-cell stage up to the late blastocyst. Neither control males nor any females showed *Rbmy1a1* expression (Fig. 2B). Consistently, RBMY1A1 protein was detectable in mutant but not control males and was never detectable in females (Fig. 2C; Supplemental Fig. S3). In line with previously described mosaic loss of imprinting (Messerschmidt et al. 2012; Lorthongpanich et al. 2013), *Rbmy1a1* expression was variable among *Trim28*^*mat*^^Δ/+^ male embryos, possibly explaining the survival of a few individuals beyond early implantation stages (Fig. 1D). In summary, loss of maternal TRIM28 results in the ectopic activation of the paternally inherited gene *Rbmy1a1* as early as the two-cell stage.

**Figure 3.**
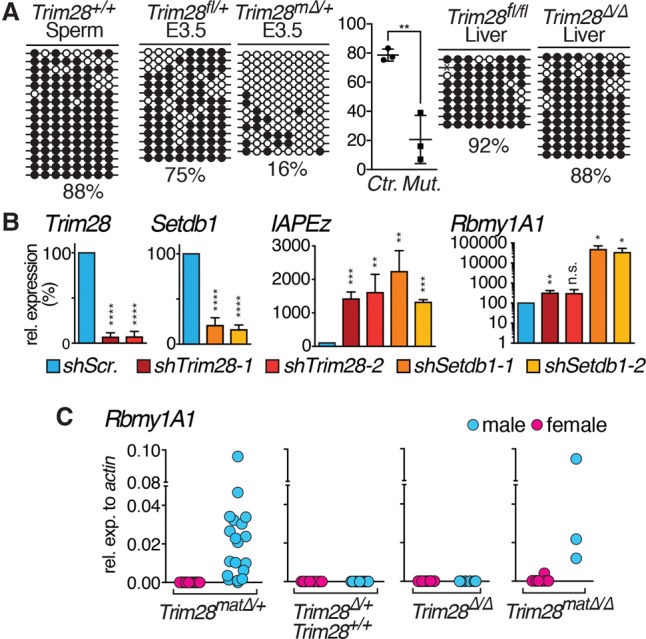
TRIM28-mediated *Rbmy1a1* repression is embryo-specific and requires maternal *Trim28* contribution. (*A*) *Rbmy1a1* promoter methylation analysis in wild-type sperm, control and *Trim28*^*mat*^^Δ/+^ E3.5 blastocysts, and control and *Trim28* knockout livers. For blastocysts, whole litters were pooled for analysis. Results for one representative litter are shown. *Rbmy1a1* promoter methylation differs significantly between control and *Trim28*^*mat*^^Δ/+^ E3.5 blastocysts (three of each. (*B*) *Trim28*, *Setdb1*, *IAPEz*, and *Rbmy1a1* (note the logarithmic scale) expression analysis after *shRNA* knockdown of *Trim28* or *Setdb1* using two independent shRNAs, respectively, in ESCs. (*C*) *Rbmy1a1* expression analyzed in male and female E3.5 blastocysts with or without maternal and/or zygotic *Trim28* contribution.

TRIM28 mediates de novo methylation and DNA methylation maintenance (Wiznerowicz et al. 2007; Quenneville et al. 2011; Messerschmidt et al. 2012; Rowe et al. 2013). We thus examined a conserved *Rbmy1a1* promoter region containing eight CpGs in sperm, embryos, embryonic stem cells (ESCs), and somatic cells (Fig 3; Supplemental Fig S4). In sperm the *Rbmy1a1* promoter is highly methylated (88%). Surprisingly, this hypermethylation is maintained in wild-type blastocysts (average 80%; *n* = 3 litters), suggesting resistance to epigenetic reprogramming post-fertilization comparable with genomic imprints. In contrast, the *Oct3/4* promoter serving as a control was fully demethylated at the blastocyst stage (Supplemental Fig. S4). However, in *Trim28*^*mat*^^Δ/+^ blastocysts, the reprogramming resistance was lost, and dramatic hypomethylation of the *Rbmy1a1* promoter ensued; only 21% of CpGs were methylated in mutant blastocysts (Fig. 3A; Supplemental Fig. S4E). This is a significant reduction compared with controls (*P* = 0.0042) (Fig. 3A) and is consistent with *Rbmy1a1* activation in *Trim28*^*mat*^^Δ/+^ male embryos. Hypomethylation was not found in hepatocyte-specific *Trim28* knockout livers (Fig. 3A) or, remarkably, *Trim28* shRNA knockdown ESCs (Supplemental Fig. S4F,G), suggesting an early embryo-specific role of TRIM28 in *Rbmy1a1* repression.

A study found that TRIM28-mediated methylation of foreign DNA is established mainly between E0.5 and E2.5 and subsequently inherited to somatic tissues (Wiznerowicz et al. 2007). Although derived from the E3.5 blastocyst, TRIM28 is still essential to endogenous retrovirus (ERV) silencing and imprinting maintenance in ESCs (Rowe et al. 2010; Quenneville et al. 2011). In most differentiated cells, with a few possible exceptions (Fasching et al. 2015), TRIM28 is dispensable for imprint maintenance and ERV silencing. Consequently, shRNA knockdown of *Trim28* in ESCs results in marked activation of *IAPEz* (Fig. 3B; Supplemental Fig. S5A,B). Surprisingly, though, *Rbmy1a1* is not substantially activated on RNA and is undetectable at the protein level (Fig. 3B; Supplemental Fig. S5A,B). Perhaps derepression alone is insufficient for *Rbmy1a1* activation due to the lack of specific transcription activators in ESCs, as shown for VL30^Pro^ elements in ZFP809 knockout cells (Wolf et al. 2015), or possibly TRIM28 may no longer be required for *Rbmy1a1* repression in ESCs, as permanent silencing has been achieved at early embryonic stages. TRIM28 mediates ERV repression through SETDB1 (Schultz et al. 2002; Matsui et al. 2010; Rowe et al. 2010), which, in contrast to TRIM28, is required for the continuous silencing of ERVs in somatic tissues (Wolf et al. 2015). In line with and in contrast to the *Trim28* knockdown, the *Setdb1* knockdown results in a very robust activation of *Rbmy1a1* in ESCs (Fig. 3B; Supplemental Fig. S5C,D). Culturing ESCs under 2i conditions to promote a naïve state did not alter these outcomes (Supplemental Fig. S5E). We thus conclude that *Rbmy1a1* can be activated in ESCs, yet TRIM28-mediated repression is restricted to the early embryo.

To test when TRIM28 is required for *Rbmy1a1* repression, we examined *Rbmy1a1* expression in blastocysts lacking maternal, zygotic, or maternal and zygotic *Trim28* (Fig. 3C; Supplemental Fig. S6). Again, *Trim28*^*mat*Δ/+^ males displayed reproducible yet variable *Rbmy1a1* activation. Control and, importantly, “zygotic” heterozygous embryos never expressed *Rbmy1a1*, the latter allowing exclusion of haploinsufficiency effects. Crucially, zygotic-null embryos did not activate *Rbmy1a1* either. The absence of maternal and zygotic *Trim28* caused variable *Rbmy1a1* activation comparable with *Trim28*^*mat*^^Δ/+^ embryos. Therefore, maternal *Trim28* alone is required and sufficient for the effective silencing of *Rbmy1a1* in the preimplantation embryo.

**Figure 4.**
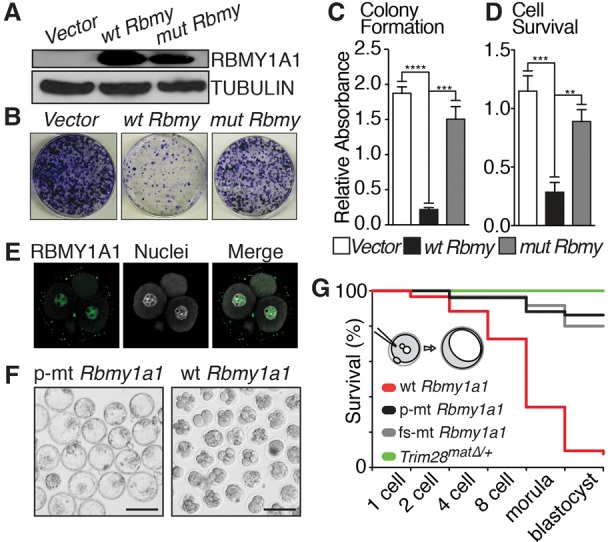
Forced *Rbmy1a1* expression hampers cell growth and embryo development. (*A*) Western blot of cells transduced with vector-only, wild-type, or mutated *Rbmy1a1*. (*B*) Images of colony formation assay using cells described in *A*. (*C*) Quantification of colony formation assay across three independent experiments. (*D*) Quantification of MMTS cell survival assay across three independent experiments. (*E*) RBMY1A1 staining of a four-cell stage embryo injected with wild-type *Rbmy1a1* mRNA. (*F*) Overview of embryos injected with mutant or wild-type *Rbmy1a1* after 4 d of in vitro culture. (*G*) Quantification of embryo survival throughout in vitro preimplantation development after injection of wild-type *Rbmy1a1* mRNA and *Rbmy1a1* mRNA variants with a five-amino-acid in-frame deletion or an early frameshift mutation. Untreated *Trim28*^*mat*^^Δ/+^ embryos show no preimplantation defects. Bars, 100 µm.

Finally, to test whether *Rbmy1a1* expression could cause the male-specific phenotype, we expressed wild-type or mutated *Rbmy1a1* in cells and embryos (Fig. 4). *Rbmy1a1* expression significantly impairs colony formation and cell viability, whereas expression of mutated *Rbmy1a1* (Supplemental Fig. S7) does not (Fig. 4B–D). Next, we injected wild-type zygotes with *Rbmy1a1* RNA variants and monitored development in vitro. Successful expression of RBMY1A1 was shown by immunofluorescence (Fig. 4E). Expression of a frameshift or in-frame deletion mutant mRNA (Supplemental Fig. S7) did not interfere with preimplantation development. Eighty percent (*n* = 48 out of 60) and 86% (*n* = 44 out of 51) of embryos developed to expanded blastocysts, respectively (Fig. 4F,G). Conversely, wild-type *Rbmy1a1* mRNA caused developmental arrest in most embryos (91%; *n* = 126 out of 138) mainly around the eight-cell to morula stage, underlining the harmful effects of RBMY1A1 outside of its endogenous (testis-specific) expression domain.

Just as for genomic imprints (Bartolomei 2009; Ferguson-Smith 2011; Messerschmidt et al. 2012; Tomizawa and Sasaki 2012; Lorthongpanich et al. 2013), we propose that maintenance of epigenetic states at yet undefined genomic loci evading epigenetic reprogramming are vital and that defects within impair development or cause disease. *Rbmy1a1* is such a novel gene, and the absence of maternal TRIM28 results in erroneous demethylation, and activation of *Rbmy1a1* causes developmental arrest. Because *Rbmy1a1* is Y-linked, the phenotype is male-specific. Remarkably, like paternal imprints (Messerschmidt et al. 2012; Lorthongpanich et al. 2013), the *Rbmy1a1* locus is exposed only to the TRIM28-null environment post-fertilization; the defect arises within the embryo. The precise timing of TRIM28 requirement is evident, as only maternal but not zygotic TRIM28 is required for *Rbmy1a1* repression. *Rbmy1a1* detected in two-cell stage mutants also supports the notion of a very early requirement of TRIM28. Furthermore, unlike other embryonic TRIM28 functions identified to date (i.e., imprint maintenance and ERV repression), *Rbmy1a1* derepression could not be recapitulated in ESCs. Finally, *Rbmy1a1* promoter hypomethylation is detectable as early as the two-cell stage (Supplemental Fig. S8A). Hence, a specialized requirement of TRIM28 at the *Rbmy1a1* locus, possibly counteracting active DNA demethylation (for review, see Messerschmidt et al. 2014) or perhaps targeting DNMTs for remethylation in the zygote (Amouroux et al. 2016), is plausible.

Retrotransposons, in particular *IAP*s, are silenced by TRIM28 in ESCs and embryos (Rowe et al. 2010). As several *IAPEz*s are found near *Rbmy1a1*, their derepression may indirectly induce neighboring *Rbmy1a1* gene copies. However, while *IAPEz*s are activated in *Trim28* knockdown ESCs and zygotic-null *Trim28* blastocysts (Fig. 3; Rowe et al. 2010), neither shows comparable activation of *Rbmy1a1.* In line with this, we could not identify *IAPEz*/*Rbmy1a1* chimeric transcripts in *Trim28*^*mat*^^Δ/+^ embryos and did not observe DNA methylation changes in an intronic *IAPEz* insertion at one *Rbmy1a1* gene copy (Supplemental Fig. S8B,C; data not shown). Thus, while we cannot exclude that minor indirect activation of *Rbmy1a1* is driven by neighboring ERVs (see weak activation in *Trim28* shRNA knockdown ESCs), the overall derepression of *Rbmy1a1* is likely *IAPEz*-independent if not fully ERV-independent.

RBMY1A1 expression in usually restricted to the testis, where it is reported to modulate splicing events (Mahadevaiah et al. 1998; Zeng et al. 2008; Liu et al. 2009). Possibly, activation of *Rbmy1a1* drives the accumulation of testis-specific or aberrant splice variants and protein products in in the embryo, ultimately triggering apoptosis. Aptly, injection of *Rbmy1a1* mRNA does not immediately impact on embryo viability, suggesting a possible disturbance in mRNA processing after embryonic gene activation. RT–PCR and cloning of *Rbmy1a1* from *Trim28*^*mat*^^Δ/+^ embryos unveiled abnormal splice variants ridden with skipped exons and alternative splice donor/acceptor sites, causing large deletions and out-of-frame truncations (Supplemental Fig. S9). Targeting its own mRNA in testis for alternative splicing (Zeng et al. 2008, 2011), these severe defects may be attributed to expression of RBMY1A1 in the unnatural embryonic environment. Indeed, analysis of RNA sequencing (RNA-seq) reads revealed a number of gene products with exon-skipping events, which was validated for three genes (*Scamp4*, *Mettl6*, and *Mybl2*) by RT–PCR in individual embryos of both sexes and genotypes (Supplemental Table 4; Supplemental Fig. S10).

All in all, our work reveals a new and unexpected genomic region critically needing protection from DNA demethylation during embryonic epigenetic reprogramming. While, in this scenario, hypomethylation triggers immediate gene activation and embryonic lethality, other yet unidentified *Trim28*-dependent regions may impact on later development or cause disease/syndromes in the adult. Our findings thus confirm the initial hypothesis and pave the way for future genome-wide studies to expose novel targets beyond the known imprinted regions and *Rbmy1a1*.

## Materials and Methods

### Mice

*Trim28*^*f/f*^ and *Zp3-cre* mice were described previously (Messerschmidt et al. 2012). *Trim28*^*f/f*^ mice were crossed with *Alb-Cre*^2^ to generate *Trim28*^*LiverKO*^ mice (Postic et al. 1999). *Tg(CAG-EGFP)D4Nagy* mice (Hadjantonakis et al. 1998) were obtained from the Jackson laboratory and crossed back to C57BL/6J. All mouse work was performed according to Institutional Animal Care and Use Committee regulations.

### Embryo isolation

Embryos were isolated as described after natural mating or superovulation (Behringer et al. 2013). For superovulation, 5 IU of PMSG and, 48 h later, 5 IU of HCG were given intraperitoneally before mating.

### Embryo mRNA injection

In vitro transcribed mRNA was diluted in water, and ∼1 pL was microinjected into the paternal pronucleus following the standard protocol (Behringer et al. 2013). Injected embryos were cultured at 37°C and 5% CO_2_ in KSOM + AA medium (Millipore).

### RNA isolation and qRT–PCR of cells and preimplantation embryos

RNA from cells was isolated using RNeasy minikit (Qiagen), and 1 µg of total RNA was used for reverse transcription. RNA from embryos was isolated using the PicoPure RNA isolation kit (Fisher Scientific) and used for reverse transcription using the High-Capacity cDNA reverse transcription kit (Applied Biosystems) using random hexamer primers. qPCR was performed using Taq-Man Fast Universal PCR master mix (Applied Biosystems) in combination with the universal probe library (Roche) on a CFX384 Touch real-time PCR system (Bio-Rad). Relative gene expression (2^ΔCt^ method) was calculated using the housekeeping gene *Actin* to normalize the target genes. The primers and probes used are listed in Supplemental Table 5.

### Single-embryo RNA-seq

Maternal mutant or control male embryos were selected for RNA isolation, reverse transcription (Smart Seq kit, Clonetech), and library preparation (Nextera XT kit, Illumina).

RNA-seq reads were mapped to the mm9 mouse genome assembly using STAR version 2.4.2a (https://github.com/alexdobin/STAR; Dobin et al. 2013) with the following parameters: --sjdbOverhang 99 --outSAMstrandField intronMotif --outSAMtype BAM Unsorted --outFilterMultimapNmax 40 --twopassMode Basic. Aligned reads were quantified for expression using Cuffdiff (version 2.2.1) (Trapnell et al. 2013). Downstream manipulation of RNA-seq results were done with in-house scripts and CummeRbund version 2.14.0. BigWig files were generated using bamCoverage from the deepTools package (http://deeptools.readthedocs.io/en/latest; Ramírez et al. 2014). Data are available online under GSE87504.

### Immunofluorescence staining of preimplantation embryos

Preimplantation embryos were stained as described previously (Messerschmidt and Kemler 2010). The antibodies and dilutions used were α-TRIM28 (1:100; Abcam), α-OCT4 (1:100; Santa Cruz Biotechnology), α-NANOG (1:100) (Messerschmidt and Kemler 2010), α-CDX2 (1:100; BioGenex), α-Caspase 3 (1:100; Cell Signaling), α-H3K27me3 (1:100; Abcam), and α-RBMY (1:100; Santa Cruz Biotechnology). Secondary antibodies (Alexa fluor 488 and 594, Invitrogen) were used at 1:250–500 dilution.

### Cell culture

E14 ESCs were cultured in DMEM with 15% ES-grade FBS (Gibco) and 1000 U/mL LIF (Millipore) or under standard 2i conditions. NIH3T3 cells were cultured in DMEM with 10% FBS. For colony formation assay, cells were seeded at a density of 1 × 10^3^ cells per 10-cm plate. Colonies were fixed and stained with 0.02% Crystal violet. Stained colonies were first photographed and then extracted in 1% SDS solution and quantified at 570 nm. For the cell viability assay (MTT), cells were seeded in 96-well plates at a density of 3000 cells per well. MTT solution was added to the cells at a final concentration of 0.5 mg/mL, and cells were incubated at 37°C before quantification.

### shRNA constructs, transfection, and lentivirus generation

shRNA oligonculeotides (Supplemental Table 5) were cloned into AgeI/EcoRI sites of pLKO.1-puro vector. Lentivirus was generated by transfection of pLKO.1 shRNA constructs with packaging and envelope plasmids into 293T cells using Lipofectamine 2000 (Life Technologies). After 24–48 h, viral supernatant was harvested, filtered, and used to transduce E14 ESCs (seeded 16–24 h earlier) along with 80 µg/mL polybrene (Sigma-Aldrich). The infected cells were selected with 2 µg/mL puromycin (Sigma-Aldrich) 48 h after infection.

### Protein isolation and Western blot analysis

Cells were lysed and extracted with RIPA buffer supplemented with protease inhibitors (Roche). Twenty micrograms of total protein was loaded onto a Tris-glycine SDS–polyacrylamide gel, separated, and transferred to nitrocellulose membrane (Bio-Rad). The membrane was blocked and then incubated with primary antibody overnight. The membrane was washed and incubated with HRP-conjugated secondary antibody before detection with chemiluminescent HRP substrate (Millipore) and exposure. The antibodies and dilutions used were α-TRIM28 (1:1000; Abcam), α-SetDB1 (1:500; Santa Cruz Biotechnology), α-α-tubulin (1:1000–5000; Sigma-Aldrich), and Rbmy1a1 (1:500–1000; Santa Cruz Biotechnology).

### Histology

Deciduomas were isolated at E6.5 and fixed in 4% PFA, processed for paraffin embedding, and sectioned. Rehydrated sections were stained with Harris hematoxylin (Sigma Aldrich) and counterstained with eosin Y (Sigma-Aldrich). Sections were dehydrated and mounted in DPX (Sigma-Aldrich).

### DNA methylation analysis

DNA methylation was analyzed by bisulfite conversion, cloning, and sequencing as described before (Messerschmidt et al. 2012). Briefly, DNA from a pooled litter of E3.5 embryos was used for bisulfite conversion according to the manufacturer's protocol (Imprint DNA modification kit, Sigma). For sperm, ESCs, or tissue samples, 1 µg of genomic DNA was used for conversion. PCR fragments were cloned and sequenced. Primers are listed in Supplemental Table S5.

### Statistical analysis

If not otherwise stated, Student's *t-*test was performed. “n.s.” indicates not significant (*P* > 0.05), single asterisks indicate *P* = 0.01–0.05, double asterisks indicate *P* = 0.001–0.01, and triple asterisks indicate *P* = 0.0001–0.001 or *P* < 0.0001.

## Supplementary Material

Supplemental Material
