# Supplementary material for: Loss of maternal Trim28 causes male-predominant early embryonic lethality
Source: Genes Dev. 2017 Jan 1;31(1):12–7. doi: 10.1101/gad.291195.116 (PMC5287108; doi:10.1101/gad.291195.116)
Supplement: Supplemental Material [file supp_31_1_12__index.html]

Loss of maternal Trim28 causes male-predominant early embryonic lethality — Loss of maternal Trim28 causes male-predominant early embryonic lethality — Supplemental Material 

# Loss of maternal *Trim28* causes male-predominant early embryonic lethality

## Supplemental Material

- Supplemental\_Tables.xlsx
- Supplemental\_Material.pdf
