# Supplementary material for: Loss of maternal Trim28 causes male-predominant early embryonic lethality
Source: Genes Dev. 2017 Jan 1;31(1):12–7. doi: 10.1101/gad.291195.116 (PMC5287108; doi:10.1101/gad.291195.116)
Supplement: Supplemental Material [file supp_gad.291195.116_Supplemental_Material.pdf]

Supplemental Information for

‘Aberrant epigenetic reprogramming causes male-specific lethality’

Sampath *et al.*

Supplemental Material and Methods:

Splice variant analysis:

To determine differential splicing events, MATS 3.0.9 beta (http://rnaseq-mats.sourceforge.net/) was used to count junction reads and reads falling into the tested region within ENSEMBL gene definitions. Splicing events were labeled significant if the sum of the reads supporting a specific event exceeded ten reads, and FDR < 0.05. Downstream analysis and graphs were generated with in-house scripts.

For RT-PCR analysis and verification of splice variants *Trim28*$^{f/f}$*; Zp3-cre* or *Trim28*$^{f/f}$ (no *cre*) females were crossed with X-GFP males. Blastocysts were isolated at E3.5 and sorted for gender based on GFP expression. RNA was isolated from blastocysts as described in the main materials and methods section. Primers are listed in Supplemental table 5. A 2% agarose gel was used to visualize PCR products. Band intensities were measured in ImageJ normalized to background levels before PSI (percentage splicing inclusion) calculation. PSI is calculated as: Inclusion (%) = [Exon(s) included / (Exon(s) included + Exon(s) skipped)] x 100.

Supplemental Figure Legends:

Supplemental Figure S1. Absence of maternal *Trim28* causes male-predominant early embryonic lethality. (A) Zygotic-null embryos are obtained from heterozygous intercrosses. 25% of progeny are expected to be *Trim28* null, 50% heterozygous, 25% wild-type. Maternal-null embryos are obtained from mating *Trim28*$^{f/f}$*; ZP3-cre* female mice to wild-type males. All progeny is maternal-null. Normal embryos inherit maternal gene products, which are replaced by embryonic gene products after embryonic gene activation (EGA) from the 2-cell stage onwards. Classic (zygotic) knock-out embryos have unperturbed, normal maternal contribution yet lack embryonic *Trim28*. Maternal-null embryos lack maternal gene products, yet activate expression from the paternal allele after EGA. (B) Representative uterus of a female carrying maternal-null embryos. Five resorbed embryos are found next to five developing embryos at E13.5 (red and green arrowheads, respectively). All developing embryos in this uterus were determined to be female. (C) Morphologically variant maternal-null E4.5 littermates showing NANOG (green) and CDX2 (red) expression. (D) Representative embryos for morphological classification applied in Figure 1G.

Scale bars: 50µm

Supplemental Figure S2. Sex-specific GFP reporter mice. (A) An ubiquitously active X Chromosome-linked GFP (X-GFP) inherited from the father allows efficient identification of X-GFP-positive female and X-GFP-negative male progeny. (B) A different pair of maternal-null littermates at E4.5 as shown in Figure 1E. The Female embryo (GFP-positive and characteristic dotted H3K27me3 staining on condensed X Chromosome) displays normal morphology while GFP-negative male mutant (without characteristic dotted H3K27me3 staining and condensed X Chromosome) shows severe defects. (C) Control female and male

littermates showing no apparent defects. (D) Representative X-GFP-positive female mutant E6.5 embryo. Mosaic X Chromosome-inactivation is apparent at this stage showing contribution of both X-GFP-positive (paternally inherited X Chromosome) and -negative cells (maternally inherited X Chromosome) to the embryo proper.

Scale bars: 100µm

Supplemental Figure S3. Representative embryos stained for RBMY1A1 at E4.5. Control (A) and mutant females (B) females lack a Y chromosome and don't stain positive for RBMY1A1 (negative control). (C) An additional morphologically aberrant *Trim28 v*maternal-null male embryo displaying nuclear RBMY1A1 expression as shown in Figure 2C.

Scale bars: 100µm

Supplemental Figure S4. DNA-methylation analysis in embryos and embryonic stem cells. (A-C) DNA-methylation analysis of the *Oct4* promoter in (A) control, (B) maternal-null *Trim28* embryos and (C) E14 ESCs. (D) Three individual control litters analyzed for *Rbmy1a1* promoter methylation. Litter 1 is shown representatively in Figure 4. (E) Three individual *Trim28* maternal-mutant litters analyzed for *Rbmy1a1* promoter methylation. Litter 3 is shown representatively in Figure 4. (F-G) DNA-methylation analysis of the *Rbmy1a1* promoter in (F) scrambled or (G) *Trim28*-shRNA knock-down ESCs.

Supplemental Figure S5. *Rbmy1a1* expression *Trim28 or Setdb1* shRNA knock-down embryonic stem cells. (A) Western blot analysis for TRIM28 and RBY1A1 protein in lysates of control (scrambled) and two independent *Trim28* shRNA-knock down cells. α-Tubulin serves as loading control. (B) Immunofluorescence analysis of IAP and RBMY1A1 expression in scrambled and a *Trim28* shRNA-knock down cell line. (C) Western blot analysis for

SETDB1 and RBY1A1 protein in lysates of control (scrambled) and two independent *Setdb1* shRNA-knock down cells. α-Tubulin serves as loading control. (B) Immunofluorescence analysis of IAP and RBMY1A1 expression in scrambled and one *Setdb1* shRNA-knock down cell line. (E) Same experiment as conducted in Figure 3 (B) yet under 2i culture conditions. Knock-down of *Trim28* using two independent shRNAs does not allow for *Rbmy1a1* activation. In contrast knock-down of *Setdb1* using two independent shRNAs results in robust activation of *Rbmy1a1*. ESC culture condition (FCS *vs.* 2i) does not change *Trim28/Setdb1* shRNA-knock down outcomes.

Supplemental Figure S6. Generation of embryos with diverse combinations of maternal and/or zygotic *Trim28* contribution. (A-D) Mating schemes used to obtain (A) maternal-null mutant, (B) wild-type/zygotic heterozygous mutant, (C) wild-type/zygotic heterozygous mutant/zygotic homozygous mutant, (D) maternal-null mutant/maternal-zygotic-null mutant embryos. (E-F) Illustration of TRIM28 protein contribution throughout pre-implantation development in the diverse embryos generated in mating schemes shown in A-D.

Supplemental Figure S7. *Rbmy1a1* variants used for overexpression experiments. (A) Alignment of *mus musculus, rattus norvegicus and homo sapiens* RBMY amino acid sequences. Highlighted in red are five amino acids (four of which are 100% conserved) predicted for RBMY function, which were deleted for the '*p-mt-Rbmy1a1*' mRNA variant. (B) Wild-type, full-length protein sequence (C) *p-mt-Rbmy1a1* protein sequence and (D) *fs-mt-Rbmy1a1* protein sequence expressed in cells and embryos for experiments shown in Figure 4. Red amino acids in (B) were deleted in *p-mt-Rbmy1a1*' mRNA variant (C, red dashes), purple labels out of frame amino acids and premature translation stop after frame shift mutation (D, *fs-mt-Rbmy1a1*)

Supplemental Figure S8. (A) DNA-methylation analysis of the *Rbmy1a1* promoter in 2-cell stage embryos. >200 maternal mutant or control embryos were pooled for bisulfite conversion respectively. Maternal-null 2-cell stage embryos show substantial hypomethylation compared to controls. (B) Schematic representation of the intronic insertion of an *IAPEz* element in one *Rbmy1a1* gene copy (chrY: 3,326,875-3,345,401; GRCm38/mm10). Black arrowheads indicate primer location used for DNA amplification after bisulfite conversion. The forward primer is located within the intronic, the reverse primer within the *IAPEz* sequence allowing locus-specific amplification. (C) DNA-methylation analysis of the 'intronic' *IAPEz* as shown in (A). Two wildtype and three maternal mutant litters were analyzed at E3.5. No statistical significant differences were found in methylation levels.

Supplemental Figure S9. Analysis of *Rbmy1a1* splice variants and defects in maternal mutant E3.5 embryos. (A) Schematic representation of *Rbmy1a1* fragment (Exon 6 to 10) RT-PCR amplification (Primers shown as red arrows). *Rbmy1a1* is only detectable in maternal-null males, not females or controls. Next to the expected fragment length (white arrowhead) multiple additional bands were detected. (B) Full-length coding sequence (CDS) was amplified from maternal-null E3.5 embryos, cloned and sequenced. In addition to the predominant full-length sequence multiple additional and aberrant splice-variants were detected. White boxes indicate intact exons. Red boxes indicate incomplete exons due to alternative splice donor or acceptor site use. Yellow boxes indicate intron sequence inclusion due to alternative splice donor or acceptor site use. Primers binding to beginning and end of coding sequence (CDS) were used (red arrows), untranslated regions (UTRs) were not analyzed.

Supplemental Figure S10. Splicing defects in *Trim28* maternal mutant male embryos. (A-C) Splicing defects found in three transcripts *Mettl6* (A), *Mybl2* (B) and *Scamp4* (C) are shown. Schematic view of the respective exon-intron structure of the gene (top) shows analyzed mis-splicing event (red line), followed by sashimi plots of one representative control and maternal mutant male embryo (middle) and confirmation by semi-quantitative RT-PCR in five individual male embryos of each genotype (bottom left). Graphs show PSI (percentage splice inclusion) quantification of analyzed bands (white arrow - normal transcript; black arrowhead - exon-skipping transcript; gels for female embryos are not shown). Splicing differences are variable yet only significantly changed in maternal-null male embryos.

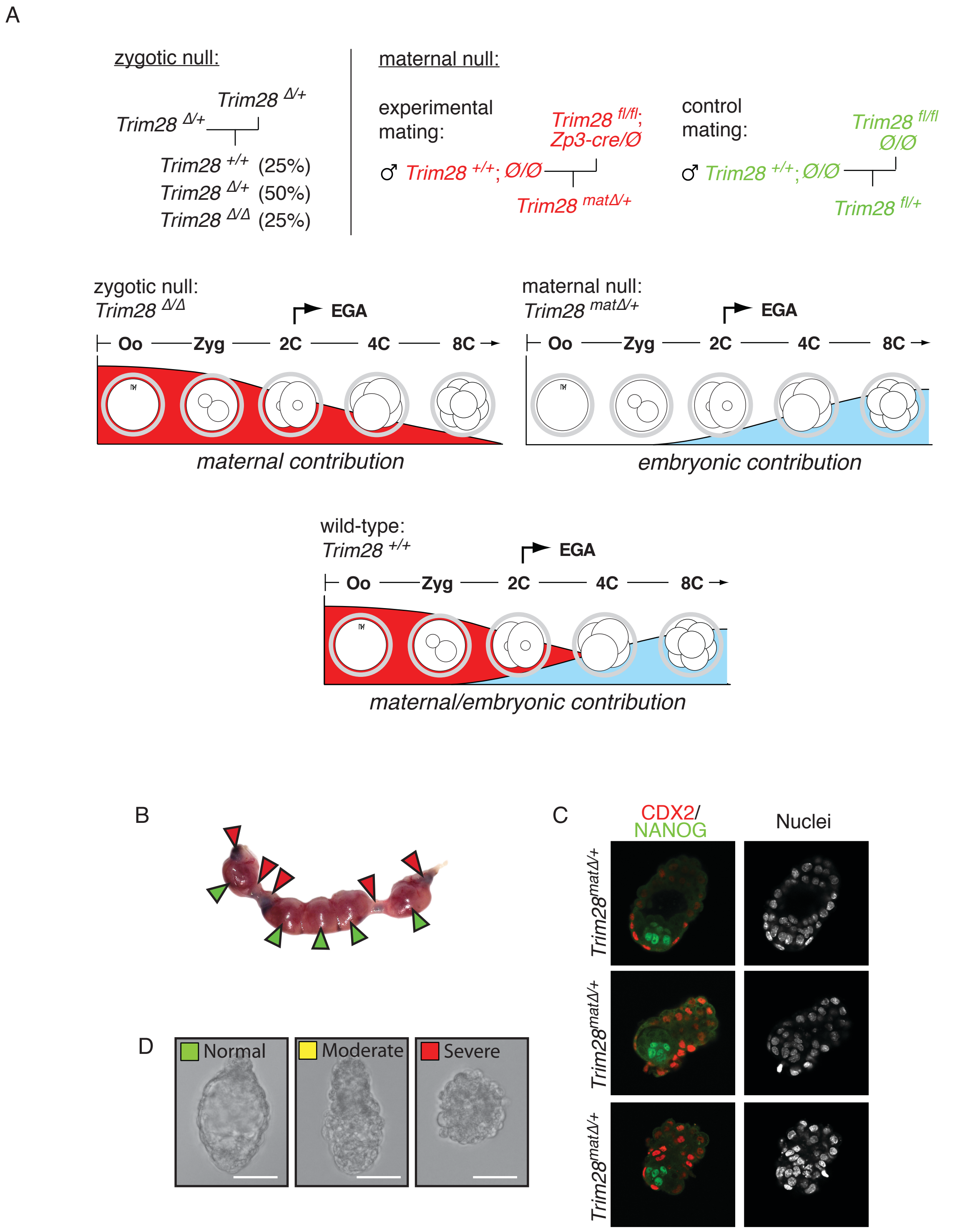
A
zygotic null:
Trim28 Δ/+
Trim28 Δ/+
Trim28 +/+ (25%)
Trim28 Δ/+ (50%)
Trim28 Δ/Δ (25%)
maternal null:
experimental mating:
Trim28 fl/fl; Zp3-cre/Ø
♂ Trim28 +/+; Ø/Ø
Trim28 matΔ/+
control mating:
Trim28 fl/fl Ø/Ø
♂ Trim28 +/+; Ø/Ø
Trim28 fl/+
zygotic null: Trim28 Δ/Δ
EGA
Oo Zyg 2C 4C 8C
maternal contribution
maternal null: Trim28 matΔ/+
EGA
Oo Zyg 2C 4C 8C
embryonic contribution
wild-type: Trim28 +/+
EGA
Oo Zyg 2C 4C 8C
maternal/embryonic contribution
B
C
CDX2/
NANOG
Nuclei
Trim28matΔ/+
Trim28matΔ/+
Trim28matΔ/+
D
Normal
Moderate
Severe

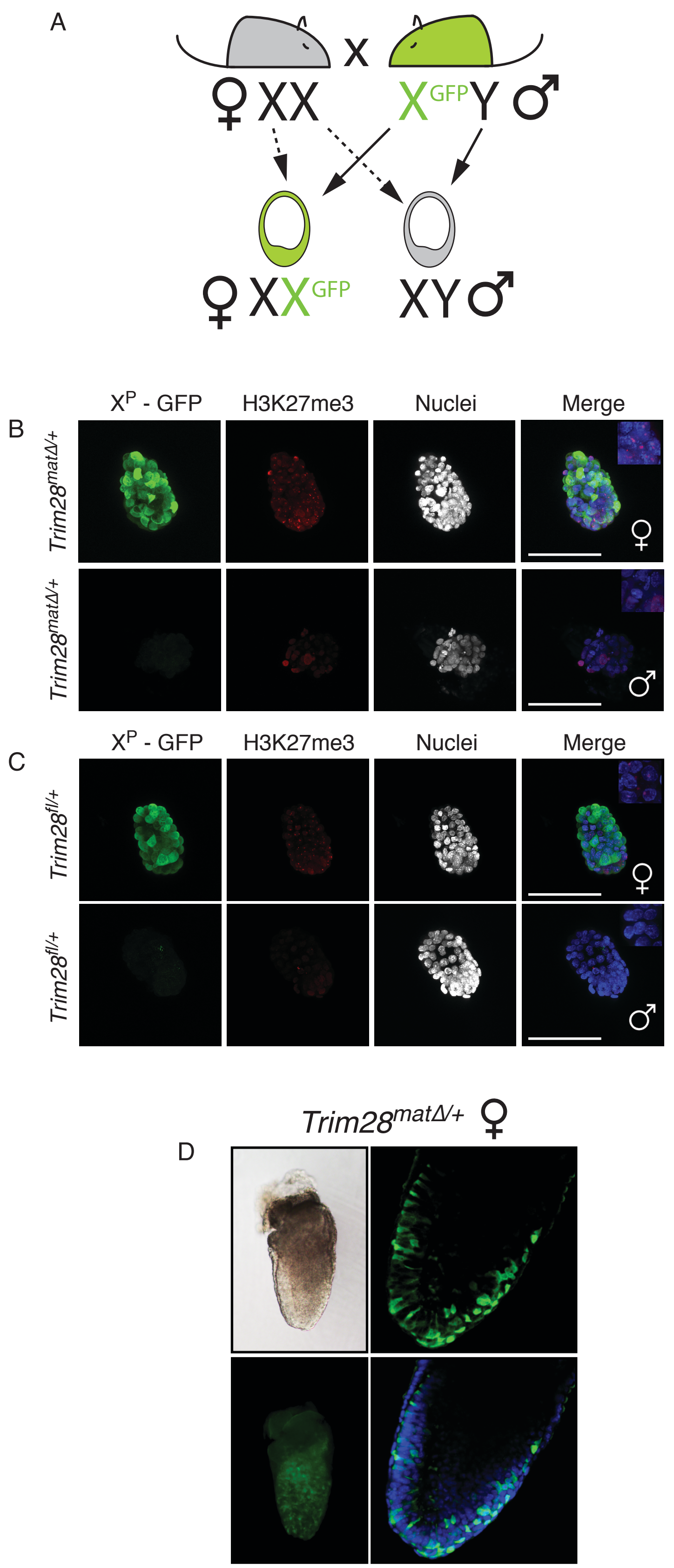
A
♀ XX
x
X^{GFP}Y ♂
♀ XX^{GFP}
XY♂
B
X^P - GFP
H3K27me3
Nuclei
Merge
$Trim28^{mat\Delta/+}$
♀
$Trim28^{mat\Delta/+}$
♂
C
X^P - GFP
H3K27me3
Nuclei
Merge
$Trim28^{fl/+}$
♀
$Trim28^{fl/+}$
♂
$Trim28^{mat\Delta/+}$ ♀
D

GENESDEV/2016/291195 - Sampath et al. Fig. S3

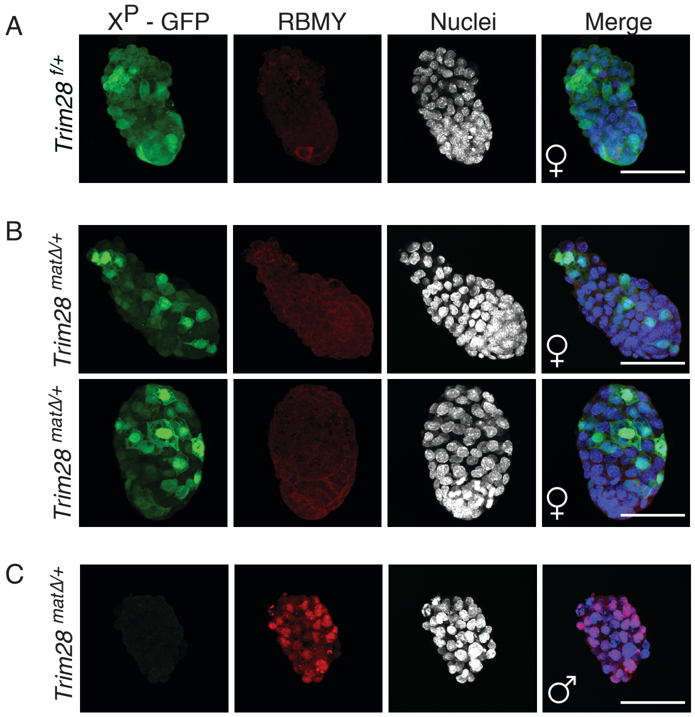

GENESDEV/2016/291195 - Sampath et al. Fig. S4

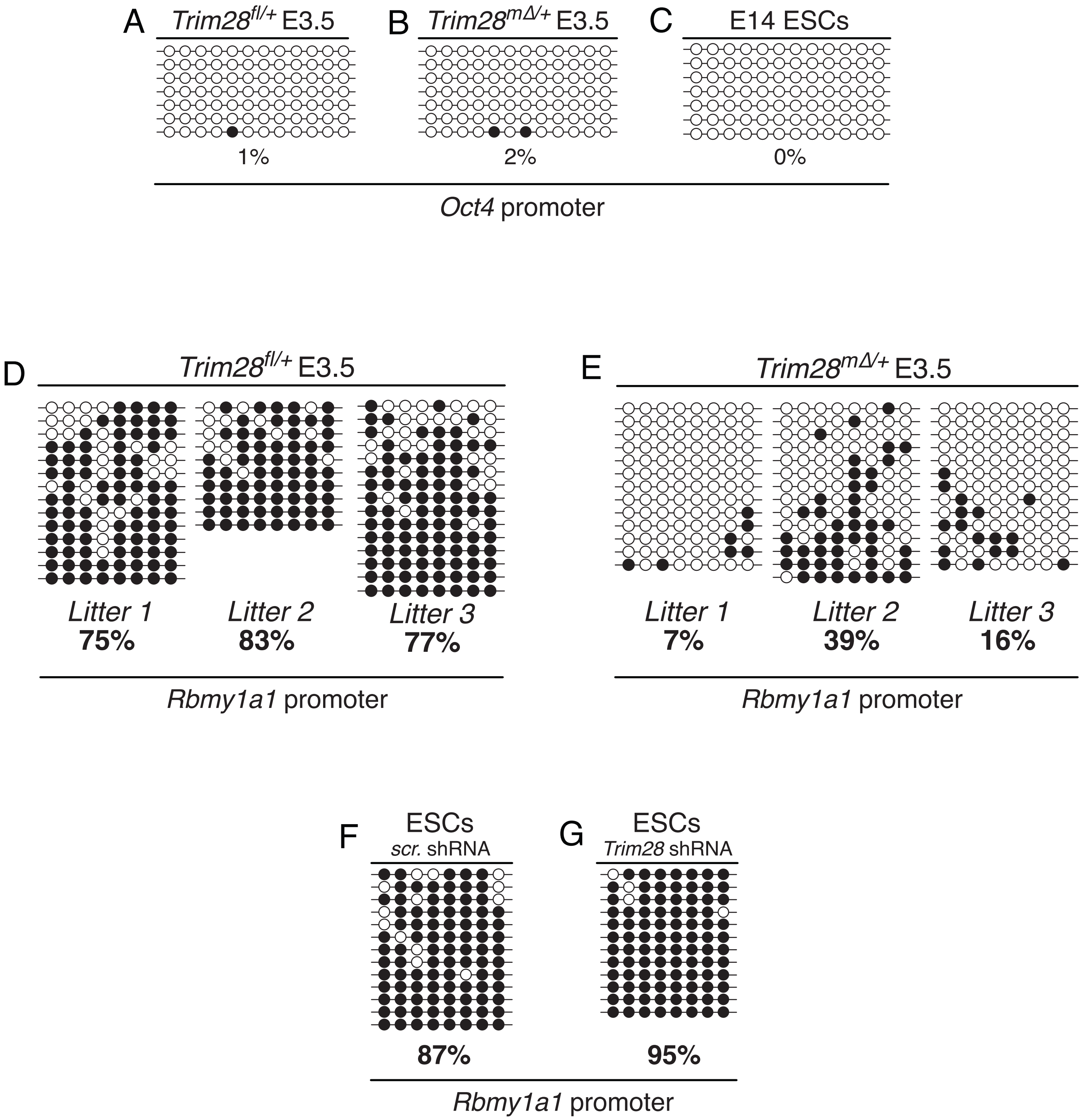

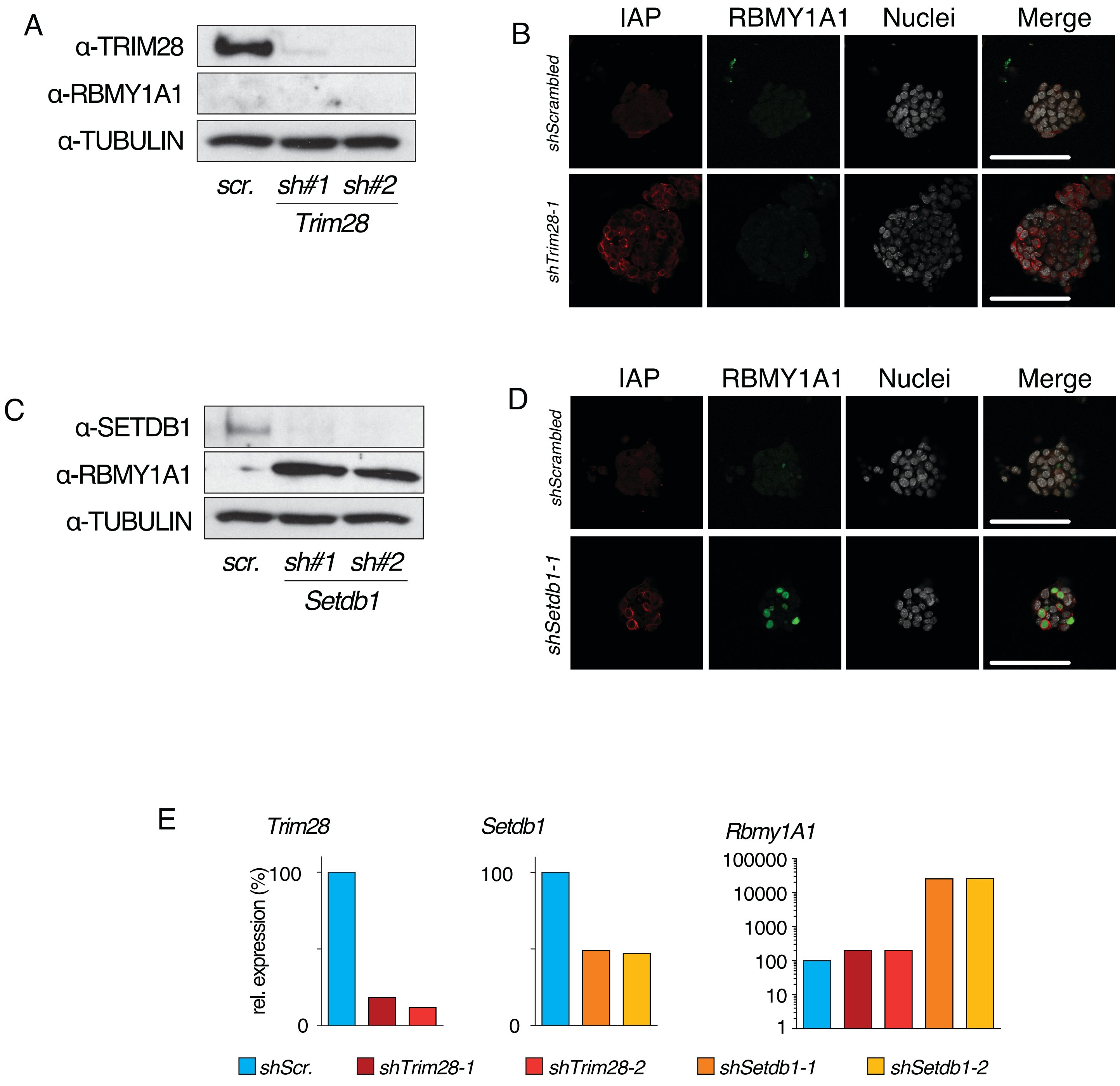
A
α-TRIM28
α-RBMY1A1
α-TUBULIN
scr.
sh#1
sh#2
Trim28
B
IAP
RBMY1A1
Nuclei
Merge
shScrambled
shTrim28-1
C
α-SETDB1
α-RBMY1A1
α-TUBULIN
scr.
sh#1
sh#2
Setdb1
D
IAP
RBMY1A1
Nuclei
Merge
shScrambled
shSetdb1-1
E
Trim28
Setdb1
Rbmy1A1
rel. expression (%)
100
0
100000
10000
1000
100
10
1
shScr.
shTrim28-1
shTrim28-2
shSetdb1-1
shSetdb1-2

GENESDEV/2016/291195 - Sampath et al. Fig. S6

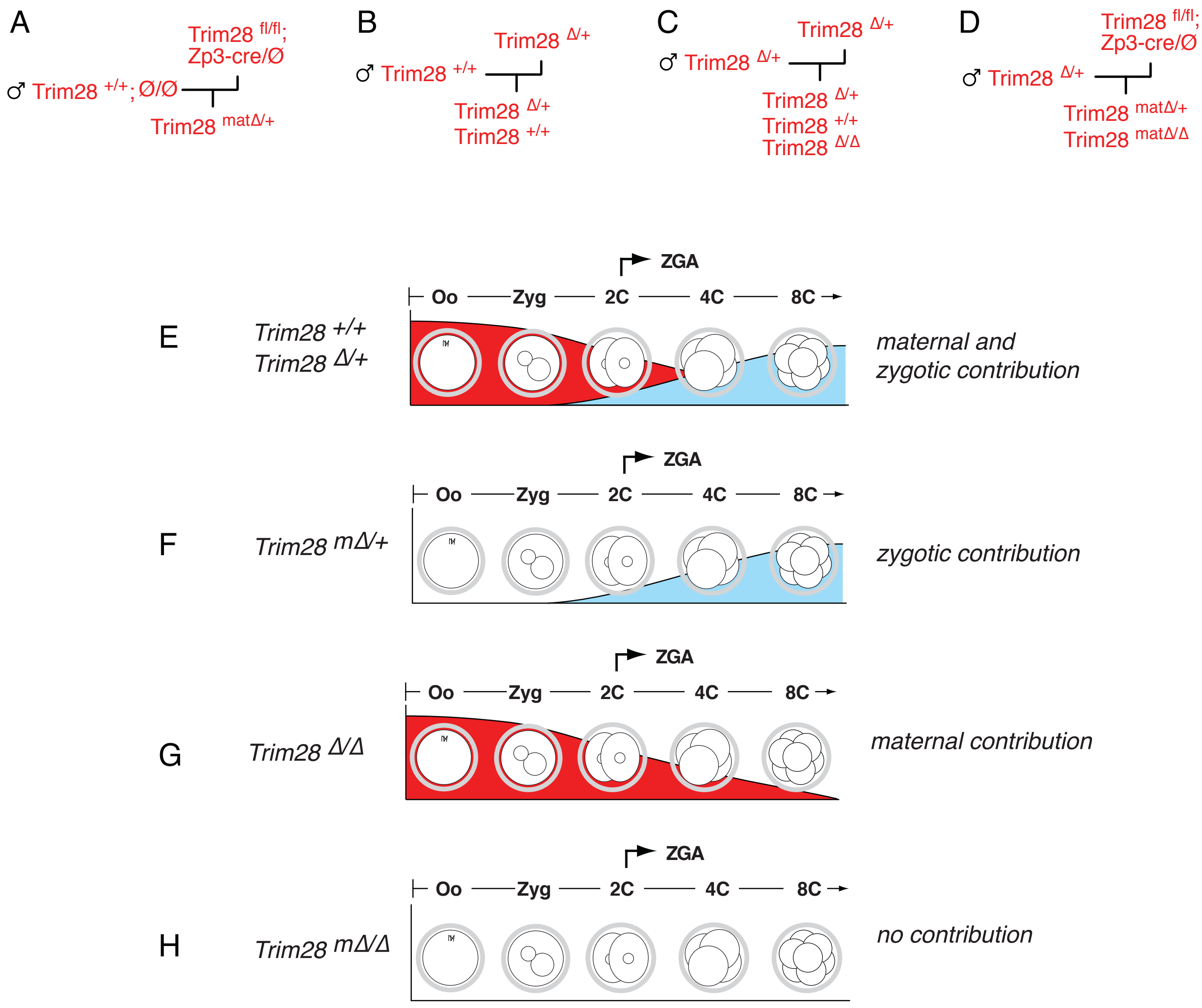

A

```
Majority               MAEXXQPGKIFIGGLNXETRQKTLQXIFGRFGPIXXVILMRDRETKKSRGFAFXTFXXPADAKNAVKEMNGVSLDGKRIKVEQARRPSSLESGSKRRPPS

Mus Musculus           MAETNQPGKIFIGGLNIKTRQKTLQEIFGRFGPVARVILMRDRETKKSRGFAFLTFRRLADAKNAVKEMNGVILDGKRIKVKQARRPSSLESGSKKRPPS 100
Rattus Norvegicus      MAESEQPGKIFIGGLNLETRQKTLQVIFGRFGPITHVILMRDRETKKSRGFAFVTFQCPGDAKNAVKEMNGVSLDGKRIKVEQARRPSSLERSSKRRPLS 100
Homo Sapiens           MVEADHPGKLFIGGLNRETNEKMLKAVFGKHGPISEVLLIKDR-TSKSRGFAFITFENPADAKNAAKDMNGKSLHGKAIKVEQAKKPS-FQSGGRRRPPA  98

Majority               FSRTRGASRILKCGRGGRSRXR-SXPSXEGNLDDGRYTPNFNXSSSGRHFAVKRXPSSKRXGPPSKRSATSAXTRSN--XGLRGREPHRREXSRNMPRRE

Mus Musculus           FSRTRGASRILKCGRGGRSRAR-SGPSCEGNLGGDRYTPNFNVSSSGRHFAVKRNPSSKRDDPPSKRSATSAQTRSN--TGLRGREPHRREISRNMPRGE 197
Rattus Norvegicus      FSRTRGASRILKCGRGGSSRGR-SRPSRERNLDDGRYTPNFNISSSGRHFAVKRSTSSKREGPPSKRSATSALTRAN--PGLRGREPHGREVSRNMPRRE 197
Homo Sapiens           SSRNRSPSGSLRSARGSRGGTRGWLPSQEGHLDDGGYTPDLKMSYSRGLIPVKRGPSSRSGGPPPKKSAPSAVARSNSWMGSQGPMSQRRENYGVPPRRA 198

Majority               PXSSRRDEYPLPRDDGYSSNDX------XXRXYASTSRGYXDYGN-YSSRDEHASKVFSDH-------------------------------AGYXGGRD

Mus Musculus           PASSRRDEYPLPRDYGQSSND---------RKYESTSRGYCDYGN-YHSREESASKVFSDH-------------------------------AGYLGGRD 256
Rattus Norvegicus      PVSSRRDEYPLPRDDGYSSNDS------YSRRYASTSRGYRDYGN-YSSRDEHASKVFSDH-------------------------------AGYHGGRD 259
Homo Sapiens           TISSWRNDRMSTRHDGYATNDGNHPSCQETRDYAPPSRGYAYRDNGHSNRDEHSSRGYRNHRSSRETRDYAPPSRGHAYRDYGHSRRDESYSRGYRNRRS 298

Majority               -RDFSEYLS---GXSYRDT-----YESYGR-FHEXPSARGT-------------XXGNNRYDDYSSS----XDGYG------------------------

Mus Musculus           -RDFSEYLS---GNSYRDT-----YRSYGR-FHEAPSARG---------------GNNRYDDYSNS----QDGYG------------------------ 302
Rattus Norvegicus      -RDFSEYLS---GSSYRDT-----YESYGS-FHEEPSARGT------------YTGNNRYDDYSSS----RGGYG------------------------ 308
Homo Sapiens           SRETREYAPPSRGHGYRDYGHSRRHESYSRGYRNHPSSRETRDYAPPHRDYAYRDYGHSSWDEHSSRGYSYHDGYGEALGRDHSEHLSGSSYRDALQRYG 398

Majority               ---------GXREXYXGSRSXIYSXXYERSGR-QEVLPPXIDREYXDRE---GRXERGHSPXDRLYXASRESYXSSSXXXGIXXGXEXXXXXSXXXRX

Mus Musculus           ---------GRGEPYISNRSNIYSSDYERSGR-QEVLPPPIDREYFDRE---GRQERGHSPKDGLYSASRESYSSNTKIWGIP-------WRSWRKQI   380
Rattus Norvegicus      ---------GGRETYSGSRSDIYG--YERSGR-Q-VLPP-IDREYSERG---GREERGHSPMDRLYRASHESYTISSRFGGYHGGHEETRYESGGRR   388
Homo Sapiens           TSHGAPPARGPRMSYGGSTCHAYSNTRDRYGRSWESYSSCGDFHYCDREHVCRKDQRNPPSLGRVLPDPREAYGSSSYVASIVDGGESRSEKGDSSRY   496
```

B

MAETNQPGKIFIGGLNIKTRQKTLQEIFGRFGPVARVILMRDRETKKSRGFAFLTFRRLADAKNAVKEM
NGVILDGKRIKVKQARRPSSLESGSKKRPPSFSRTRGASRILKCGRGGRSRARSGPSCEGNLGGDRYTP
NFNVSSSGRHFAVKRNPSSKRDDPPSKRSATSAQTRSNTGLRGREPHRREISRNMPRGEPASSRRDEYP
LPRDYGQSSNDRKYESTSRGYCDYGNYHSREESASKVFSDHAGYLGGRDRDFSEYLSGNSYRDTYRSYG
RFHEAPSARGGNNRYDDYSNSQDGYGGRGEPYISNRSNIYSSDYERSGRQEVLPPPIDREYFDREGRQE
RGHSPKDGLYSASRESYSSNTKIWGIPWRSWRKQI.

C

MAETNQPGKIFIGGLNIKTRQKTLQEIFGRFGPVARVILMRDRETKKSRGFAFLTFRRLADAKNAVKEM
NGVILDGKRI-----RRPSSLESGSKKRPPSFSRTRGASRILKCGRGGRSRARSGPSCEGNLGGDRYTP
NFNVSSSGRHFAVKRNPSSKRDDPPSKRSATSAQTRSNTGLRGREPHRREISRNMPRGEPASSRRDEYP
LPRDYGQSSNDRKYESTSRGYCDYGNYHSREESASKVFSDHAGYLGGRDRDFSEYLSGNSYRDTYRSYG
RFHEAPSARGGNNRYDDYSNSQDGYGGRGEPYISNRSNIYSSDYERSGRQEVLPPPIDREYFDREGRQE
RGHSPKDGLYSASRESYSSNTKIWGIPWRSWRKQI.

D

MAETNQPGKIFIGGLNIKTRQKTLQEIFGRFGPVARGNSLKETLF.

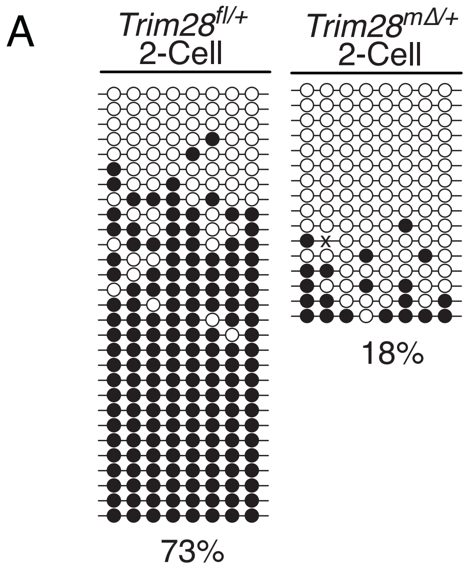
A
Trim28^fl/+
2-Cell
Trim28^mΔ/+
2-Cell
73%
18%

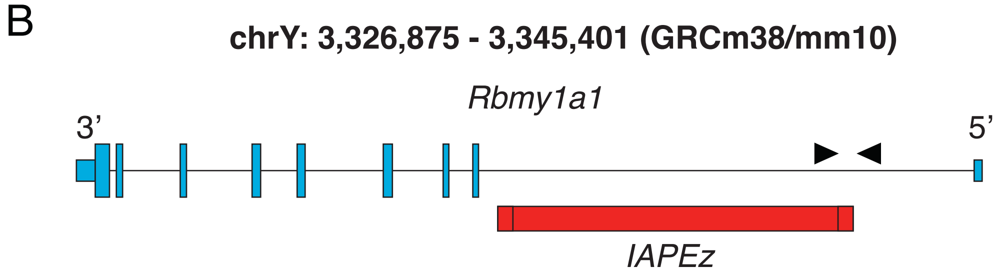
B
chrY: 3,326,875 - 3,345,401 (GRCm38/mm10)
Rbmy1a1
3'
5'
IAPEz

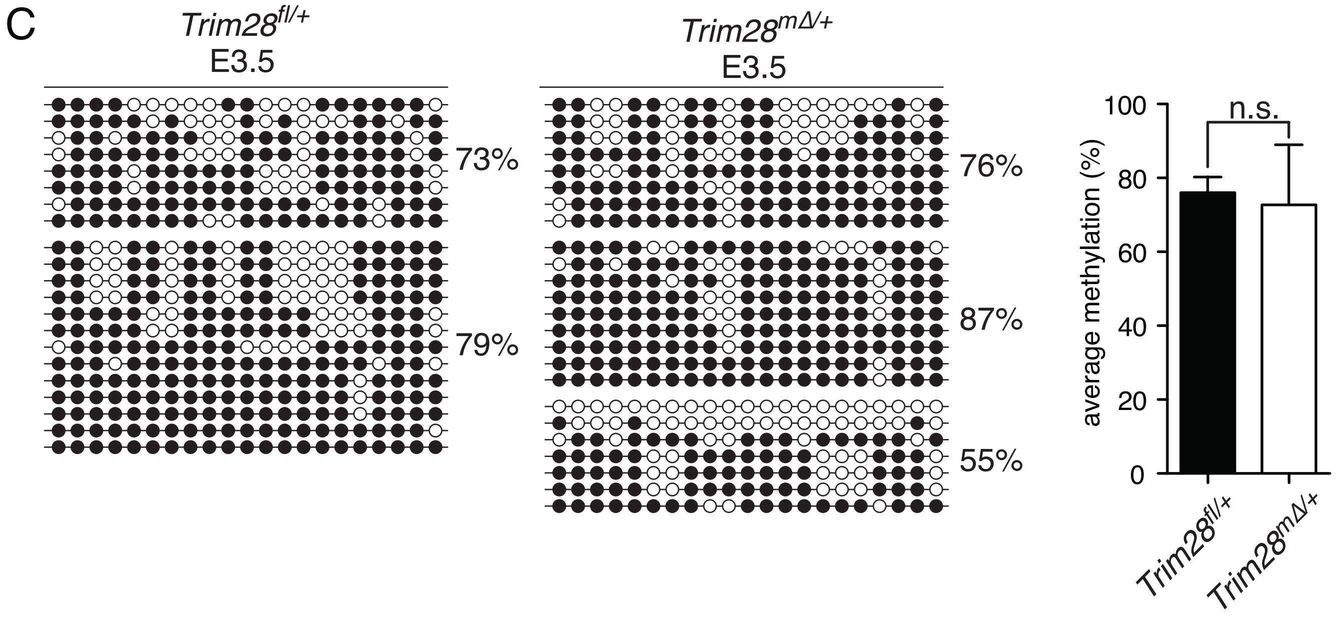
C
Trim28^fl/+
E3.5
Trim28^mΔ/+
E3.5
73%
79%
76%
87%
55%
average methylation (%)
100
80
60
40
20
0
n.s.
Trim28^fl/+
Trim28^mΔ/+

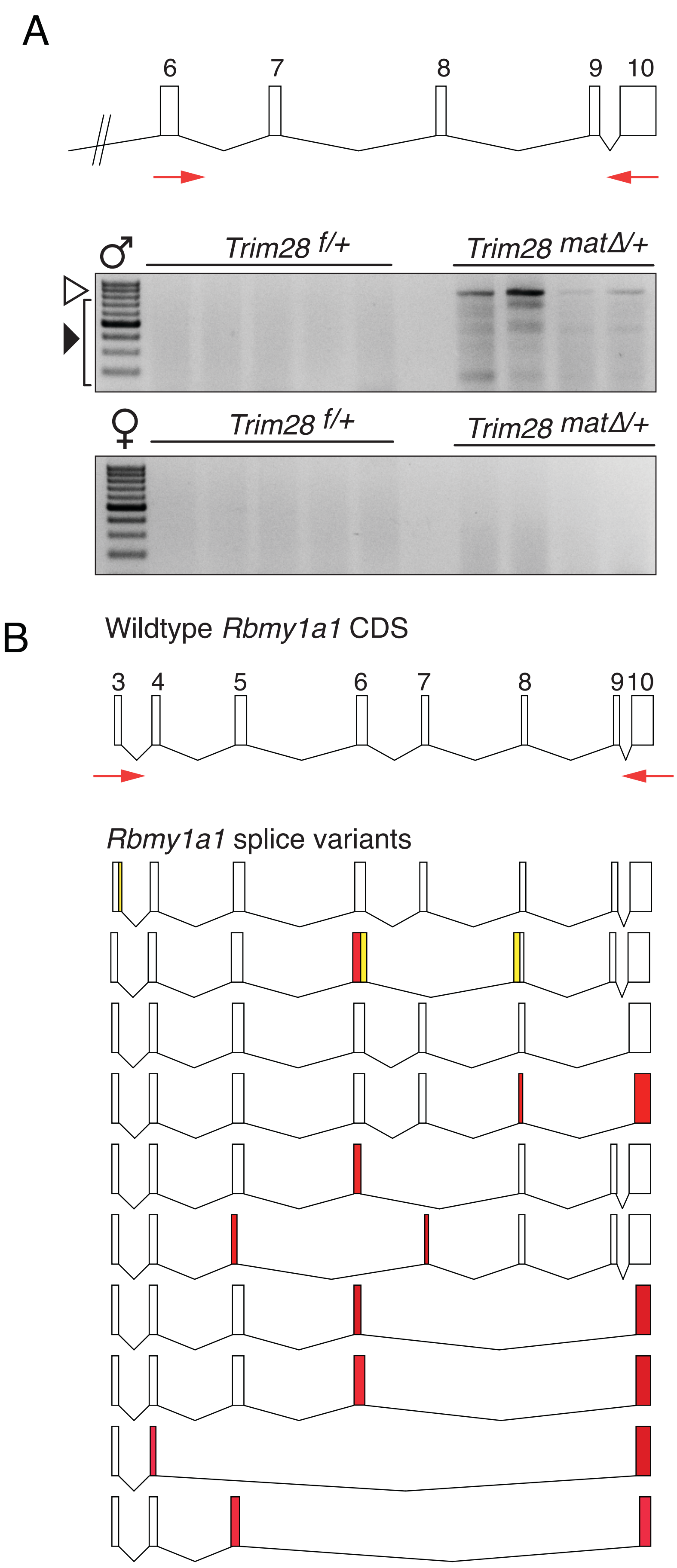
A
6
7
8
9
10
Trim28 f/+
Trim28 matΔ/+
Trim28 f/+
Trim28 matΔ/+
B
Wildtype Rbmy1a1 CDS
3
4
5
6
7
8
910
Rbmy1a1 splice variants

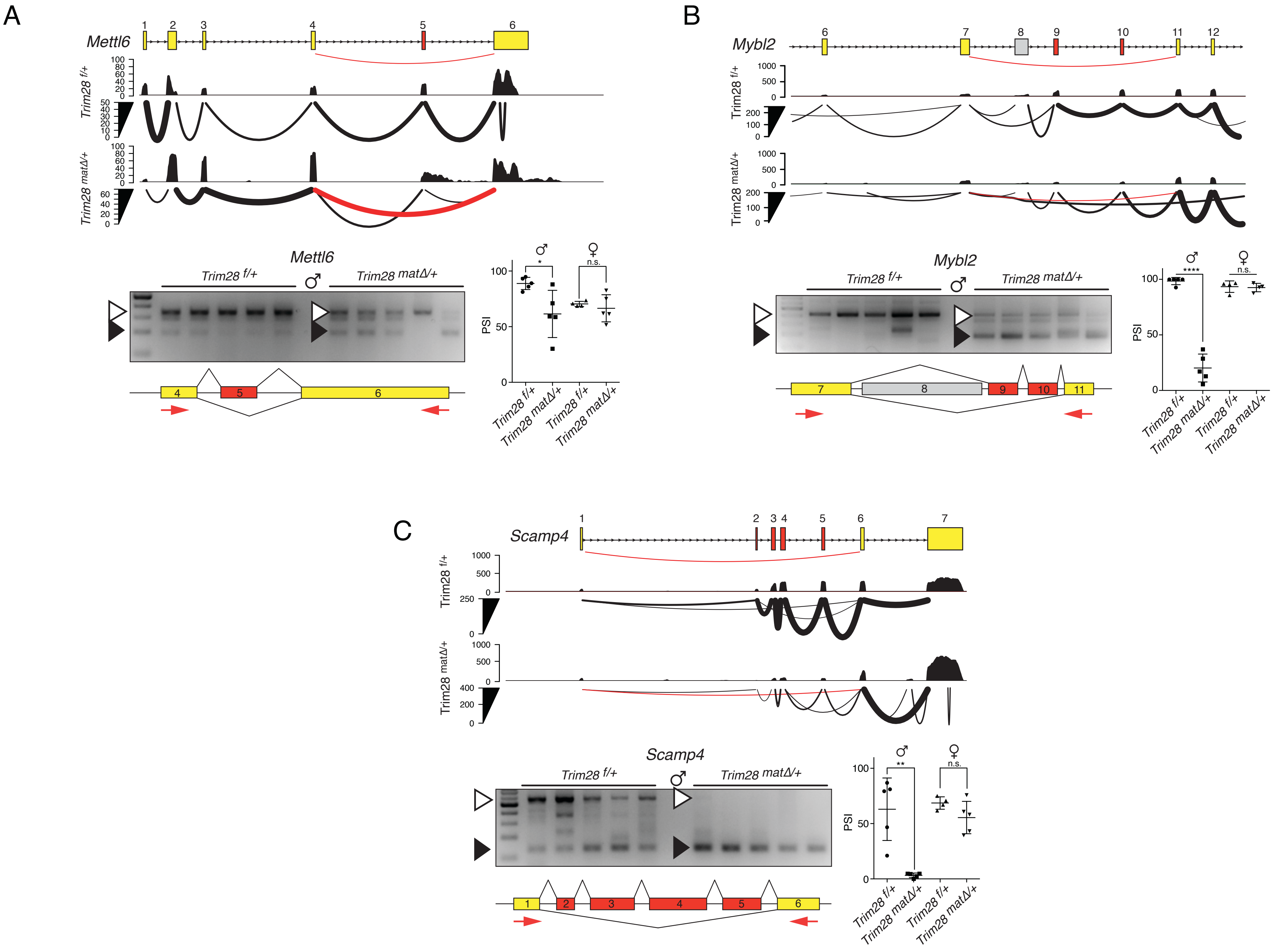
A
Mettl6
Trim28 f/+
Trim28 matΔ/+
Trim28 f/+
Trim28 matΔ/+
PSI
n.s.
B
Mybl2
Trim28 f/+
Trim28 matΔ/+
PSI
****
n.s.
C
Scamp4
Trim28 f/+
Trim28 matΔ/+
PSI
**
n.s.
